# Healthy Parent Carers peer-led group-based health promotion intervention for parent carers of disabled children: protocol for a feasibility study using a parallel group randomised controlled trial design

**DOI:** 10.1186/s40814-019-0517-3

**Published:** 2019-11-23

**Authors:** Gretchen Bjornstad, Kath Wilkinson, Beth Cuffe-Fuller, Katharine Fitzpatrick, Aleksandra Borek, Obioha C. Ukoumunne, Annie Hawton, Mark Tarrant, Vashti Berry, Jenny Lloyd, Annabel McDonald, Mary Fredlund, Shelley Rhodes, Stuart Logan, Christopher Morris

**Affiliations:** 1 Peninsula Childhood Disability Research Unit (PenCRU) and National Institute for Health Research Applied Research Collaboration South West Peninsula (PenARC), University of Exeter Medical School, University of Exeter, St. Luke’s Campus, Heavitree Road, Exeter, EX1 2LU UK; 2 Nuffield Department of Primary Care Health Sciences, Medical Sciences Division, Radcliffe Observatory Quarter, University of Oxford, Woodstock Road, Oxford, OX2 6GG UK; 3 National Institute for Health Research Applied Research Collaboration South West Peninsula (PenARC), University of Exeter Medical School, University of Exeter, St. Luke’s Campus, Heavitree Road, Exeter, EX1 2LU UK; 4 Health Economics Group, University of Exeter Medical School, University of Exeter, St. Luke’s Campus, Heavitree Road, Exeter, EX1 2LU UK; 5 PenCRU Family Faculty, University of Exeter Medical School, University of Exeter, St. Luke’s Campus, Heavitree Road, Exeter, EX1 2LU UK; 6 Exeter Clinical Trials Unit, University of Exeter Medical School, University of Exeter, St. Luke’s Campus, Heavitree Road, Exeter, EX1 2LU UK

**Keywords:** Behaviour change, Well-being, Resilience, Peer support, Patient and public involvement, Disabled children, Parents, Carers

## Abstract

**Background:**

Parent carers of disabled children are at increased risk of mental and physical health problems. They often experience challenges to maintaining good health which have implications for their well-being and their ability to care for their children. In response to these needs, researchers and parent carers developed the Healthy Parent Carers (HPC) programme. It is a peer-led, group-based intervention that promotes behaviours associated with health and well-being. The aims of this trial are to assess the acceptability of the HPC programme and the feasibility of its delivery in the community and to assess the feasibility and acceptability of the design of the definitive trial to evaluate the programme’s effectiveness and cost-effectiveness.

**Methods:**

We will establish six research sites and train facilitators to deliver the manualised intervention. Parent carers of children with special educational needs and disabilities will be individually randomised, stratified by group delivery site, to either take part in a group programme and online resources (intervention) or to receive access to the online resources only (control). Measures of mental health; well-being; health-related quality of life; health behaviours; patient activation; protective factors such as resilience, social connections, and practical support; and use of health care, social care, and wider societal resources will be collected before randomisation (baseline), immediately post-intervention, and 6 months later. Recruitment of participants, adherence to the programme, and the dose received will be assessed. Group sessions will be audio-recorded to evaluate the fidelity of delivery and participant engagement. Participants’ and facilitators’ feedback on the programme content and delivery, their experience, and the acceptability of the outcome measures and trial design will be collected through feedback forms, interviews, and focus groups.

**Discussion:**

This trial will assess whether the programme delivery and evaluative trial design are feasible, to inform whether to progress to a definitive randomised controlled trial to test the effectiveness and cost-effectiveness of the Healthy Parent Carers programme.

**Trial registration:**

ISRCTN, ISRCTN151144652, registered on 25 October 2018; ClinicalTrials.gov, NCT03705221, registered on 15 October 2018.

## Background

There are an estimated 960,000 disabled children in the UK, which is 7.3% of the population of children aged 0–18 years [1]. Parent carers of disabled children commonly report higher levels of stress and depression [2–11] and poorer physical health [3, 4, 7, 8, 12–14] than parents of typically developing children. Population-based studies suggest these health problems persist and may worsen over time [15]. These problems have implications for their ability to care for their children.

Parent carers often find the demands of caregiving have a negative impact on their physical and emotional health. Nevertheless, it is important to acknowledge that not all parents of disabled children report that their child’s difficulties negatively affect their psychological or physical health [5], and in fact, some report positive impacts [8, 16]. Indeed, some parent carers may perceive a high burden with looking after a child with a relatively ‘mild’ condition whereas others, whose child may have more severe disabilities, may not perceive caring as high a burden [4].

Some interventions target external factors, such as navigating healthcare services [17], while others target levels of stress [18, 19] or emotional and social support [20]. A systematic review of psychological therapies for parents of children with chronic illness suggested promising results in terms of improved parent mental health, particularly for problem-solving therapy [21]. No benefits were found for cognitive behavioural therapy or family therapy on parent outcomes; however, the quality of the evidence was low and analyses were limited by lack of data available to the reviewers. A systematic review of mindfulness interventions for parents of children with autism indicated potentially positive effects on parents’ stress levels and psychological well-being, with studies reporting good attendance and retention in 8-week programmes [22]. There is growing evidence that groups can facilitate change processes beneficial to health and well-being [23, 24] by enabling the formation of strong psychological connections and/or social identification with other group members which can enhance engagement, and thus possibly increase the interventions’ effectiveness [25, 26].

The idea for this research came directly from parent carers who had been involved in a study evaluating peer support for parent carers [20]. They wanted to extend the benefits of emotional support to specific strategies to improve health and well-being. Researchers and parent carers in the Peninsula Childhood Disability Research Unit (PenCRU) Family Faculty co-created the Healthy Parent Carers (HPC) programme [27].

Previously, we tested the principle and acceptability of a 6-week intervention programme with one group of seven parent carers, delivered by the intervention developers. The intervention was developed using Intervention Mapping [28] and extensive stakeholder engagement and is described in detail in a separate paper [29]. Participants had children with various conditions including autism, cerebral palsy, and acquired brain injury. Retention of participants in our preliminary study was high with all staying until the end of the 6-week programme and 2-month follow-up. Participants’ and facilitators’ feedback were positive, indicating the intervention was feasible to deliver and acceptable to, and valued by, participants. The intervention content and delivery methods were refined following feedback, and the manual was updated.

This feasibility trial will provide information that will be used to determine whether to progress to a definitive trial of the HPC programme, which would have the following objectives:

1. To determine whether the peer-led, group-based HPC programme is more effective at improving health and well-being compared to providing online information only
2. To estimate the costs of delivering the HPC programme, and the cost-effectiveness of the programme, versus the provision of online information
3. To understand how the Healthy Parent Carers intervention is working, for whom, and in what context to inform the implementation of the programme should it be shown to be effective

The current trial aims to assess the acceptability of the HPC programme and the feasibility of its delivery in the community, as well as the feasibility and acceptability of the design of the definitive trial in order to evaluate whether a fully powered randomised controlled trial is warranted and to determine the optimal trial design.

## Methods

### Objectives

This trial has two overarching aims:

1. To evaluate whether the programme can be delivered in the community by facilitators other than the developers, specifically to:
  - Assess the feasibility of establishing venues, and identifying and training group facilitators to be in a position to deliver the intervention
  - Assess the fidelity of intervention delivery in terms of format, content, and quality
  - Assess the experience and engagement of participants, facilitators, and trainers
  - Assess the programme attendance
2. To provide information necessary to design a definitive randomised controlled trial, specifically to:
  - Assess the feasibility of recruiting participants in different sites
  - Assess the acceptability of randomisation of parent carers
  - Assess the attrition and completion and proportion of any missing data in questionnaire measures
  - Appraise the performance of candidate health and well-being outcome measures in terms of acceptability to participants, and feasibility and interpretability for researchers
  - Estimate the variability (standard deviation) and the level of clustering within programme delivery groups in the intervention arm to help inform the sample size calculation for the definitive trial
  - Test the proposed cost-effectiveness framework for a future randomised trial

This will help inform whether to progress to a definitive randomised controlled trial to test the effectiveness and cost-effectiveness of the programme and provide information necessary to design the trial.

### Design

**Fig. 1:**
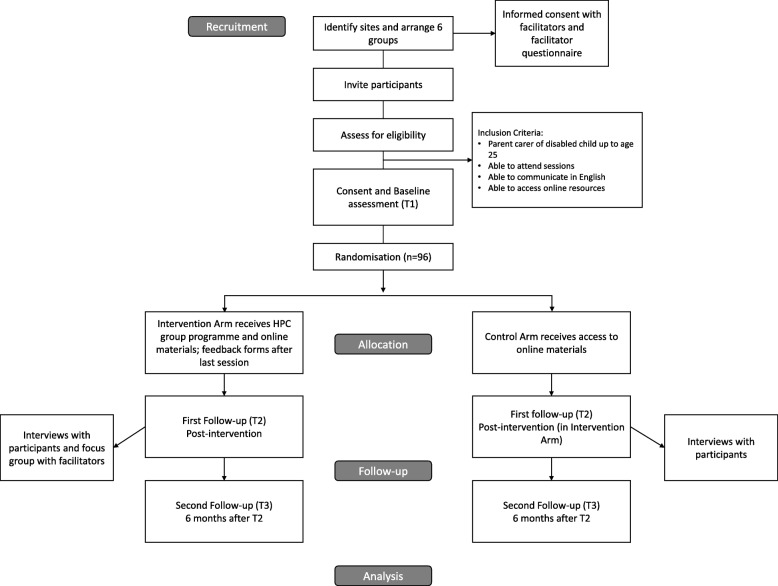
Trial flow chart

A feasibility trial using a parallel group randomised controlled trial design will be carried out in six sites in the southwest of England. Participants will be randomly allocated to receive the group-based programme and access to online programme resources or to a control group receiving access to the online resources only. Data collection will take place at three time points in both trial arms at baseline (prior to randomisation), immediately post-intervention, and 6 months later. As the intervention can be delivered over 6 or 12 weeks, the post-intervention data collection time point will vary relative to randomisation but will be consistent in terms of the amount of time passing after completion of the intervention. Participants in the control arm in each randomised site will complete measures at the same time as participants in the intervention arm for that site. The two arms will therefore be balanced in terms of the timing of outcome measures. The trial design and the flow of participants through the trial are illustrated in the trial flow chart and SPIRIT figure (Fig. 1 and Table 1). The SPIRIT checklist is provided as an additional file (see Additional file 1).

**Table 1:**
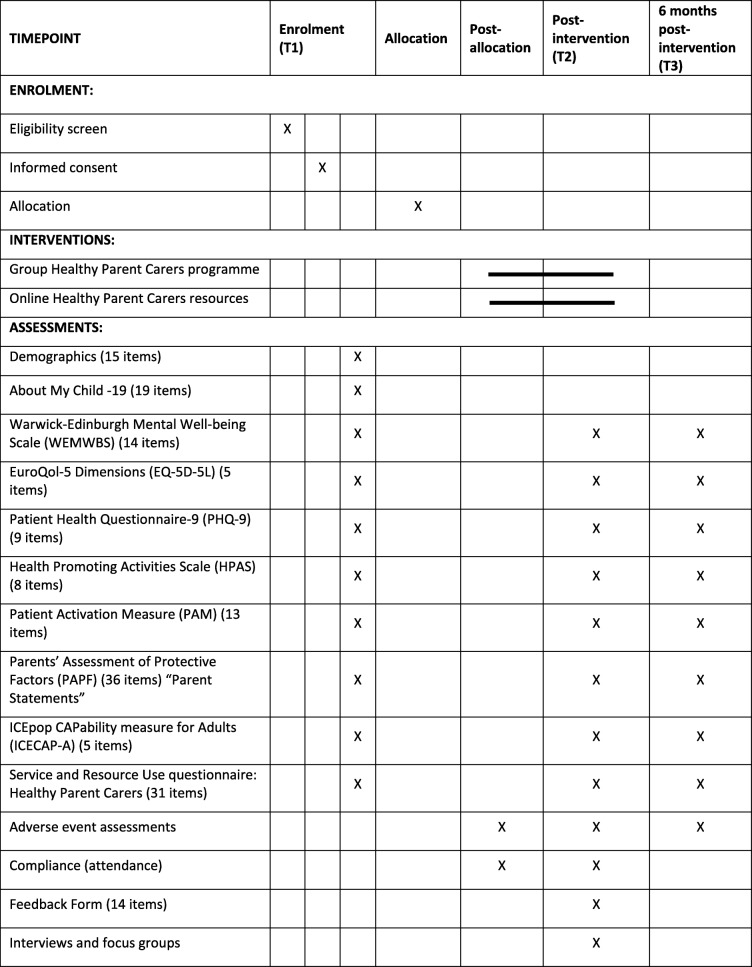
Schedule of enrolment, interventions, and assessments

### Public involvement

This project has a strong ethos of parent carer and stakeholder engagement from inception. The public involvement in this project will ensure the following: (a) the research is conducted in an acceptable manner, (b) the research outputs are relevant and useful to parents of children with special educational needs and disabilities, and (c) our dissemination materials and methods are appropriate and accessible.

Over 40 parents of children with a range of conditions from the PenCRU Family Faculty public involvement group have participated in a study-specific working group since 2014. Our Stakeholder Advisory Group (SAG) includes representatives from the local authority, public health, parent carer forums, relevant charities, and special schools.

Parent carers have been involved in all stages of developing the intervention and designing the feasibility trial including:

- Proposing the idea for the project based on their needs and experiences
- Co-designing and refining the intervention and training content and delivery methods
- Providing feedback on research methods including the selection of the comparison conditions
- Advising on the content and form of the Resource Use Questionnaire for use as part of the cost-effectiveness framework
- Contributing to interpreting and disseminating the findings of the previous study
- Interviewing and hiring research staff
- Discussing and advising on the design of the feasibility trial
- Recommending responses to peer reviews when applying for funding.

### Study setting and location

In collaboration with our SAG, we will identify six venues (e.g. schools, community centres, adult and community learning venues) where it is possible to establish and host a group. We will agree days, times, durations and frequencies of sessions, and local named organisers for each venue.

### Sample size

We aim to recruit 96 participants, to be allocated on 1:1 ratio to intervention and control. This is a large enough sample to estimate the percentage providing data at follow-up (assumed to be 80%*—*76 participants) with a margin of error of 10 percentage points based on the lower bound of the 95% confidence interval. Assuming that 38 participants are followed up in each trial arm*,* this will be large enough to estimate the standard deviation for continuous outcomes in each arm within 29% of its true value based on the upper bound of the 95% confidence interval. Finally, 76 participants at follow-up *are* large enough to estimate a correlation coefficient of 0.5 between baseline and follow-up scores for a continuous outcome with a margin of error of 0.19 based on the lower bound of the 95% confidence interval. We will randomise a minimum of *8* and *a* maximum of 24 participants at each of the 6 sites. This will mean that 4–12 participants will be allocated to each of the intervention and control trial arms at each site. We expect the ideal group size to be between 6 and 12 people but are allowing for potential attrition and variation in recruitment between sites.

### Inclusion criteria

People meeting the inclusion criteria are (1) primary carers of children with additional needs and/or disabilities (participants who self-identify as primary carers are eligible; the child can be up to 25 years old consistent with the current Department of Health and Department of Education Special Educational Needs and Disability (SEND) legislation in England and The Children’s Act; no named diagnosis is necessary, and we are not limiting to specific conditions), (2) willing and able to attend the programme group meeting session(s) on arranged dates/times, and (3) able to access online information.

### Exclusion criterion

Potential participants who are not able to communicate in English are excluded. This is necessary because the programme has not yet been translated into other languages.

### Recruitment

We will advertise the study in several ways. Press releases and interviews in local television and/or radio will be used to publicise the study. Members of our SAG will be asked to advertise the project to their members through their email lists and networks. We will also share study adverts and information via social media and through the PenCRU Family Faculty email list, asking *the* members to share in their networks. We will liaise with Information and Advice Services in each locality, staff in social services, and Special Educational Needs Coordinator *(*SENCOs*)* in schools in each study site to target potentially more isolated parent carers. We will also recruit participants at events for parent carers at the study venues and other venues in the southwest where interested parent carers can discuss the project with the research team. This recruitment strategy uses many different approaches because parent carers do not all access the same services and not all parent carers are connected with their local parent carer forums. We will ask all participants how they heard about the study during screening*,* and this information will be recorded to assess whether some recruitment methods may be more effective than others. However, we are mindful that while some methods may not result in large numbers of recruits, they may help us to reach parent carers who are more isolated and more in need of support and, as such, will be seen as important methods to take forward in a definitive trial.

Interested parent carers will contact the researchers. There will be *a* telephone or face-to-face screening to check *the* eligibility, understanding of the study*,* and to answer any questions. A researcher will meet each potential participant individually. Those who want to participate will sign a consent form and complete baseline questionnaires online using an electronic patient-reported outcome (ePRO) system with a researcher on hand for support as necessary [30]. Reasons for not consenting to participate will be recorded if provided by those who decline.

### Allocation to trial arms

When recruitment is completed at each site, we will proceed to randomisation. Each of the six programme groups will constitute a study site, with participants who choose that group being randomised to either attend the group or receive the online resources only. A computer-generated randomisation sequence will be used to assign the participants in each site to the intervention and control arms. A block randomisation scheme will be implemented to ensure balance in the number of participants allocated to each trial arm, stratified by group delivery site. The allocation sequence will be concealed from researchers using an online central randomisation service setup and maintained by the Exeter Clinical Trials Unit (UKCRC Registration ID 65). Blinding will not be used in this trial.

All participants will receive an email and letter indicating the result of randomisation. The participants randomly allocated to the intervention arm will be sent details of the group sessions and be contacted by their lead facilitator before the first group session. Participants in both arms will receive a link to the online programme resources and instructions on the web page. We will monitor the number of participants who refuse participation and record their reasoning (if they wish to share it) to gauge the acceptability of our trial design.

### Intervention

The group-based programme was developed using Intervention Mapping approach [28]. Full details of the intervention, including its development, logic model, and content (e.g. activities, behaviour change techniques), are available in a previous publication [29]. In brief, the programme aims to expand parent carers’ social networks and provide social support from peers with a shared sense of social identity alongside targeted activities to improve parent carers’ confidence, motivation, self-efficacy, and empowerment, thus creating the conditions for change necessary for them to feel able to make their own plan to prioritise healthy behaviours for themselves.

The programme content is based around a set of universal and evidence-based actions (called CLANGERS) associated with health and well-being. CLANGERS stands for Connect, Learn, be Active, Notice, Give, Eat well, Relax and Sleep [31]. The ‘CLANG’ component comprises the ‘Five Ways to Wellbeing’ based on the evidence from the foresight project on Mental Capital and Wellbeing [32]. Each of these behaviours is potentially more difficult for parent carers.

The programme content is organised into 12 modules lasting 2 h each. The modules can be delivered weekly over 6 sessions (comprising 2 modules per session) or 12 sessions (1 module per session). Our Family Faculty PPI working group suggested that offering either 6 longer sessions in the daytime or 12 shorter evening sessions would be reasonable for most parent carers. One or both options will be offered per area in order to maximise recruitment and to reflect likely real-world delivery in community settings, with 6 groups being delivered in the study in total. If uptake is very low for a particular site, the delivery model may be adapted during recruitment to increase numbers.

### Facilitators

The lead facilitators of the group-based programme will be experienced facilitators of the ‘Expert Parent Programme’ courses created by the Council for Disabled Children (CDC) with funding from the Department of Health. The facilitators are parent carers. CDC has a selection process, trains, and provides supervision for their facilitators to ensure that they facilitate the groups effectively. Their nationwide network of over 70 facilitators provides a sustainable model for the implementation of the programme in the future. Facilitators will use the Healthy Parent Carers Facilitator Manual that includes module outlines, content, timings, activities, and resources needed. The Facilitator Manual also includes safeguarding procedures for the facilitator to follow in case of any adverse events such as suicidal ideation or disclosure of safeguarding issues.

There will also be an assistant facilitator in each group to assist the facilitator in sessions. We will recruit a local parent carer for this role using a person specification detailing the required personal qualities and skills and a selection process.

We will provide training for lead and assistant facilitators. The training will take place over 4 days for lead facilitators and 2 days for assistant facilitators; it will be delivered by researchers and the parent carer co-investigators who co-developed the programme and facilitated the group in our previous study. Lead facilitators will receive ongoing supervision and support through the CDC and support from the research team. Conference calls with facilitators, assistants, and researchers will be convened to reflect on delivery of the sessions. These discussions will inform intervention design and training needs and provide a forum to share ideas and ways to address any challenges arising.

The Healthy Parent Carers online resources are part of the intervention. They reflect the content of the group sessions, provide space to write down reflections, and prompts to set specific goals and for self-monitoring of CLANGERS-related behaviours.

If the participants in the group programme miss a session, they will be telephoned by the facilitator, who will summarise the session and encourage the participant to reflect on their week, read the section of the HPC online resources, and set their weekly goals.

### Control

Participants in the control arm will receive access to the HPC programme resources online with instructions. Risk of contamination between participants allocated to each arm is low because the intervention is predicated on participants developing a shared social identity as members of the HPC group. Participants in both arms will be asked whether they have had contact with participants in the other arm of the trial as part of a post-intervention feedback form.

### Data collection

#### Study records

We will record the data on the feasibility of recruitment, including how many people respond to the adverts, how they heard about the study, reasons for not taking part for those who decline, and how many are successfully recruited. We will record delivery setting, delivery model, attendance, attrition, and reasons for missed sessions or withdrawal from the study. We will also monitor how long it takes to accrue the target number of participants at each site and at what point in the trial process any participants withdraw.

#### Sample characteristics

Demographic data will include gender, ethnicity, parent relationship status, number of children, employment status, level of education, income, housing status, and age, gender, and diagnosis (if any) of their disabled child. We will also collect information about their disabled child’s functioning and health complexity using the About my Child measure (AMC-19) [33]. We will also use participants’ postcodes to link with the Indices of Multiple Deprivation as an indicator of deprivation relative to England and Wales in the area where participants live [34].

#### Outcome measures

Participants will be asked to complete all measures before randomisation, immediately post-intervention, and 6 months post-intervention, regardless of attendance or engagement with the interventions. Based on the recommendations from our Family Faculty PPI working group, the measures will be available to complete online, using a computer, smartphone, or tablets. The Exeter Clinical Trials Unit will set up an online platform for participants to access and complete the measures. Participants may request to complete the measures on paper if they wish. Any measures completed on paper will be independently double-entered by two researchers. These requests will be monitored to track preferences for online- versus paper-based measures.

Two members of our Family Faculty PPI working group have tested the applicability and time to complete the measures (45 min). A £25 shopping voucher will be posted to participants as acknowledgement for completing measures at each time point.

The measures will comprise:

- Warwick-Edinburgh Mental Well-being Scale (WEMWBS): The WEMWBS is a 14-item scale used to assess the mental well-being in the general population and in the evaluation of programmes aiming to improve mental well-being [35]. Responses are normally distributed in the general population. WEMWBS has been validated in the UK, Europe, and elsewhere. It has been tested with minority ethnic populations, users of mental health services, and carers. It is sensitive to changes occurring through participation in programmes that promote well-being such as health promotion programmes. A tariff of well-being-adjusted life-year weights is currently being developed for responses on the WEMWBS, which will enable the measure to be used in cost-effectiveness analyses recognised by the National Institute for Health and Care Excellence (NICE) [36].
- Patient Health Questionnaire-9 (PHQ-9): The PHQ-9 is a 9-item measure that rates the frequency of symptoms and is designed for screening, diagnosing, monitoring, and measuring the severity of depression [37–39]. Categories based on the cutoff scores of 5, 10, 15, and 20 represent none, mild, moderate, moderately severe, and severe depression, respectively. As part of a safeguarding protocol, we will use the Patient Health Questionnaire (PHQ-9) to measure depressive symptoms. The PHQ-9 questionnaire is recommended by NICE to assess depression in adults [40], and its use is highlighted in clinical pathways [41], so the interpretation of scores is widely understood by GPs and primary care staff. There is good evidence, across a range of studies, for the validity, reliability, sensitivity, and specificity of the PHQ-9 for detecting depressive disorders [38, 39]. It can be administered repeatedly to assess change in depression in response to treatment. Question 9 screens for suicidal ideation. If the person scores higher than 0 on question 9, or at any other point discloses suicidal ideation, we will follow the safeguarding protocol.
- EuroQol 5 Dimensions (EQ-5D-5 L): The EQ-5D-5 L is a measure of health-related quality of life. It consists of five items measuring five dimensions of health-related quality of life (mobility, self-care, usual activities, pain/discomfort, and anxiety/depression) and a vertical visual analogue scale measuring self-rated health [42]. QALY weights can be applied to EQ-5D scores, which can then be used to calculate QALYs, and the cost-per-QALY of the intervention in a future definitive trial. The EQ-5D is NICE’s preferred measure for use in health technology cost-effectiveness analyses.
- Parents’ Assessment of Protective Factors (PAPF): The PAPF is a 36-item measure that assesses protective factors identified in the development of the Strengthening Families evidence-based parenting programme [43]. These protective factors are as follows: parental resilience, social connections, concrete support in times of need, and support of children’s social and emotional competence. These factors relate to the determinants of change in the logic model for the Healthy Parent Carers Programme.
- Health Promoting Activities Scale (HPAS): The HPAS is an 8-item measure of a person’s estimation of the frequency with which they participate in a range of activities that promote or maintain health and well-being [44, 45]. It was developed for and validated with mothers of children with disabilities.
- Patient Activation Measure (PAM): The PAM is a 13-item measure that measures the spectrum of skills, knowledge, and confidence in patients and captures the extent to which people feel engaged and confident in managing their own health and care [46, 47]. It has been tested with 100,000 patients with long-term conditions in England to establish the feasibility of using the PAM across the NHS, how activation can inform support for self-management, what support clinicians and commissioners need to use the measure effectively, and whether supporting activation can improve outcomes for patients in the NHS.
- ICEpop CAPability measure for Adults (ICECAP-A): The ICECAP-A is a 5-item measure of capability, which includes the following aspects of well-being found to be important to adults in the UK: attachment, stability, achievement, enjoyment, and autonomy [48–50]. A set of UK well-being adjusted life-year weights are available for the ICECAP-A, enabling it to be used in economic evaluations.
- Resource use questionnaire: We developed a study-specific resource use questionnaire in collaboration with parent carers. This includes health, social care, participant, and broader societal resource use, and draws on measures in the Database of Instruments for Resource Use Measurement (DIRUM) [51].

### Process evaluation

In line with the MRC guidance on process evaluations, this study will include a process evaluation that is appropriate for the feasibility testing stage of the development-evaluation-implementation process for this intervention [52]. This process evaluation will allow for the exploration of the feasibility of implementation of the intervention by assessing uptake (recruitment) and retention, participant engagement, fidelity of delivery (to content and quality), experiences of participants and facilitators, unintended consequences, and contextual factors which may influence experience and delivery.

The following data collection tools will be used to assess fidelity of intervention delivery, participant and trainer characteristics/motivations, trainer knowledge and self-efficacy to deliver the programme, participant engagement with the programme/online materials, and acceptability of the intervention and trial design.

- Facilitator pre-training questionnaire: We will use a pre-training questionnaire to collect information about facilitators’ characteristics, their motivations to take part, relevant background and experience, and expectations of delivering the programme.
- Facilitator training feedback: Following delivery of the training, we will use a questionnaire to gather the facilitators’ feedback about their self-reported knowledge, understanding, skills, and confidence to deliver the intervention and to gather their reflections on the training.
- Facilitator delivery observations: We will use a checklist to assess lead facilitators’ competence to deliver while observing their delivery of the programme content during the facilitator training. The checklist includes key skills and competencies linking to the objectives of the lead facilitator training and will enable trainers and research staff to assess facilitators’ readiness to deliver the programme. This will also help to guide and plan additional or future training.
- Facilitator checklist, records, and support calls: We will use a self-report checklist completed jointly by the facilitators to indicate which content they have covered in each session (adherence), the duration of the sessions (dose), and the participants’ engagement. Facilitators will be asked to record attendance at each session. We will also arrange support calls with facilitators to gather more information about how the groups are going and any challenges to delivery.
- Session recordings: We will audio-record group sessions and will sample two to three recordings from each group to assess fidelity to intervention content, quality of delivery, and participant engagement. A researcher will rate the delivery using the same checklist used by the facilitators after each session. A second researcher will rate one recording per group (*n* = 6, 14%). The two researchers will compare the scoring of the first three groups immediately, and any inconsistencies will be discussed with JL/MT, to ensure there is a clear understanding of the assessment criteria. The scores of the double-coded sessions will be agreed between researchers, and the sessions assessed by the researchers will be compared with the facilitators’ scores.
- Participants’ feedback: We will collect feedback from treatment and control arm participants about the programme content and delivery, their experiences, and whether they had contact with participants in the other arm of the trial via feedback forms at the end of each group session for those in the intervention arm and at the end of the programme for those in both trial arms.
- Participant interviews: We will sample purposively 12 participants (from different groups) in the intervention arm and 6 participants allocated to the control arm across all sites for semi-structured telephone interviews. For the intervention arm, these interviews will explore participants’ experiences of, and engagement in, the programme and the group and their views on the group content, activities, and facilitators. All, control and intervention groups, participants will be asked in the interviews about engagement with online resources, perceived impact of the programme and any potential contextual influences, and acceptability of the trial processes and measures. We will also sample up to 4 participants (from different groups) who were allocated to the intervention arm of the trial but did not attend any group sessions to ask them about barriers to attending and whether anything could be done to promote attendance in future groups. All interviews with participants will last approximately 30 min and will be audio-recorded and transcribed verbatim, with names and other personal identifying information changed to protect confidentiality. Interviews will take place as soon as possible after the participants have completed their post-intervention measures and before they complete their 6-month follow-up measures.
- Focus groups with facilitators: We will invite all lead facilitators and assistant facilitators to a focus group after the end of all groups. The focus group (lasting approximately 2 h) will cover facilitators’ experiences of delivering, and engagement with, the programme, views on the programme content, activities and feasibility of programme delivery, facilitator training and skills, group management, and suggestions for improvements. The focus group will be audio-recorded and transcribed verbatim (with any potentially identifiable information anonymised).

### Cost-effectiveness framework

We will develop and test a framework for assessing the cost-effectiveness of the intervention in a future randomised trial. We will:

- Establish methods for estimating intervention resource use and costs (e.g. training of facilitators, facilitators’ time, venue hire), in collaboration with the programme facilitators and site representatives
- Develop a resource use questionnaire in collaboration with parent carers, drawing on measures in the Database of Instruments for Resource Use Measurement (DIRUM) repository [51]
- Assess the acceptability to parent carers of the EQ-5D-5 L, the ICECAP-A, and the WEMWBS, judged by missing data and measurement properties [53].

### Data analysis

#### Statistical/quantitative analysis

We will report the number of eligible people who self-refer and the percentage (with 95% confidence intervals (CIs)) of these that are randomised in the trial. These findings will also be reported separately for each type of delivery setting and delivery model to assess whether particular delivery settings or models are more popular and therefore would lead to higher recruitment rates in the subsequent definitive trial. We will also report numbers and percentages of people who heard about the study via different sources, organised into categories.

We will also report the percentage (with 95% confidence intervals) of participants who complete each assessment at each time point as an assessment of the acceptability of the measures and of the feasibility of collecting sufficient data in a definitive trial. We will summarise the characteristics of recruited participants using demographic data to allow for assessment of the representativeness of the sample relative to figures available from the Office for National Statistics on the population in the southwest of England. We will also report the baseline comparability of the trial arms with respect to demographics and outcome measures.

For the intervention arm, we will report the number and percentage of participants that attend each group session with 95% confidence intervals. The percentage of participants that are lost to follow-up at each follow-up point will be reported for each trial arm. Select characteristics (parent carer gender and age, child gender and age, Index of Multiple Deprivation quintile, study site (centre), and baseline scores on outcomes) will be compared between those who are and are not lost to follow-up within each trial arm using descriptive summaries, but no formal statistical tests. Acceptability, judged by missing data of EQ-5D-5 L and ICECAP-A, will help to plan methods for estimation of cost-effectiveness in the future trial.

Means, standard deviations, and the correlation between baseline and follow-up scores on continuous outcomes will be reported to inform the sample size calculation for the subsequent definitive trial.

Level of clustering within groups in the intervention arm will be quantified using the intra-cluster (intra-group) correlation coefficient to inform the sample size calculation for the definitive trial; however, we recognise the relatively small sample size for this purpose, and it will be used alongside information about levels of clustering in published studies of trials of similar group-based interventions in similar settings.

We will compare the outcomes at follow-up between the two trial arms based on the intention-to-treat principle with participants analysed according to the trial arm they were randomised to. Missing data will not be imputed. We will report only confidence intervals for the intervention effect and no *p* values, in line with the extension to the CONSORT statement for reporting randomised pilot and feasibility studies [54].

#### Analysis of process data

Descriptive statistics will be reported for the quantitative data collected in delivery observation checklists, facilitator checklists, checklists used to assess intervention session recordings, and participant feedback forms.

Qualitative data collected from feedback forms, interviews, and focus groups will be analysed thematically to provide insights into participants’ experiences of the programme, intervention acceptability, and suggestions for improvement, and to enhance understanding of the impact of the intervention and the mechanisms of change in relation to the programme logic model [54]. Data will be analysed using inductive thematic analysis [55] separately for each data source (i.e. feedback comments, interviews, and focus groups), following the same approach. Some issues will emerge as more salient than others and the interpretation of findings will be influenced by the original research objectives as well as the themes emerging directly from the data.

NVivo software (version 12 Pro for Windows, QSR International) will be used to organise and analyse the qualitative data. Initially, two researchers will independently read and code line-by-line a sample of the data and discuss their coding to develop and agree on the coding framework. New codes may be added as the coding proceeds, and the codes and coded data will be reviewed. Codes will be defined, compared to each other, and organised into categories and themes. Attention will be paid to negative, or ‘deviant’, cases to inform developing themes and interpretation. Short summaries of each interview will be also written to explore how individual experiences and views of the intervention may differ between participants. The analysis and interpretation of the data will be regularly discussed with the research team. A detailed record will be kept of the analysis process, including definitions of the themes and concepts and their application.

## Discussion

We will interpret the findings of this feasibility trial and report the implications for progression to a definitive randomised controlled trial of the HPC programme. This will include any necessary amendments to the intervention content and delivery, as well as the development of a train-the-trainer manual to be used in training future facilitators of the programme. The following indicators of feasibility will be used to determine whether a definitive randomised controlled trial is feasible with the current trial design and procedures:

- Recruit a minimum of 48 participants, which is the minimum number that will enable all six sites to be randomised and the intervention to be tested
- Deliver 6 groups in total for the intervention arm, assessed by establishing 6 venues, and identifying and training facilitators, and groups completing the programme curriculum
- At least 80% of participants completing measures at 6-month follow-up or a clear plan to achieve this in the trial

If any of these indicators are not met, the research team will consider whether a definitive trial may not be feasible or whether changes to the design or procedures and further feasibility testing are needed. The need to translate programme materials into other languages will also be taken into account for a subsequent trial.

A complete and transparent report of the trial will be produced with reference to recommendations of the CONSORT 2010 statement: extension to randomised pilot and feasibility trials, including a CONSORT participant flow chart [54]. The report will be written for publication in a peer-reviewed, open access, academic journal with authorship eligibility determined by following the International Committee of Medical Journal Editors recommendations [56]. A plain language summary of the findings will also be co-produced with members of our Family Faculty public involvement group and sent to trial participants and organisations that help to recruit participants and host the groups. We will consult our Family Faculty and Stakeholder Advisory Group for advice on ways to disseminate the findings.

NHS England’s Commitment to Carers states ‘Helping carers to provide better care and to stay well themselves will contribute to better lives for those needing care and more effective use of NHS resources’ [57]. However, there is currently a paucity of interventions that promote health for parent carers. This feasibility trial and a subsequent definitive trial may have important implications for a public health strategy for parent carers of children with disabilities in the UK. It will also inform research and public health policy internationally, as the higher risk of psychological and physical health problems in parent carers is not limited to the UK.

### Project timetable and milestones

The main milestones are as follows. Ethical approval for the study was received on 20 August 2018. The trial was registered on 25 October 2018 (ISRCTN 15144652). Recruitment of participants began on 29 October 2018. The analysis of data on fidelity and process evaluation will be conducted following data collection (summer to autumn 2019). The analyses of outcome measures will be conducted in February 2020. The expected date of completion is June 2020.

## Supplementary information

**Additional file 1.** SPIRIT 2013 Checklist: Recommended items to address in a clinical trial protocol and related documents*.

## Data Availability

Not applicable

## Abbreviations

AMC-19: About my Child measure
CDC: Council for Disabled Children
CLANGERS: Connect, Learn, be Active, Notice, Give, Eat well, Relax, and Sleep
CONSORT: Consolidated Standards of Reporting Trials
DIRUM: Database of Instruments for Resource Use Management
EQ-5D-5 L: EuroQol 5 Dimensions
HPAS: Health Promoting Activities Scale
HPC: Healthy Parent Carers
ICECAP-A: ICEpop CAPability measure for Adults
NIHR: National Institute for Health Research
PAM: Patient Activation Measure
PAPF: Parents’ Assessment of Protective Factors
PenCLAHRC: The National Institute for Health Research (NIHR) Collaboration for Leadership in Applied Health Research and Care (CLAHRC) South West Peninsula
PenCRU: Peninsula Cerebra Research Unit
PHQ-9: Patient Health Questionnaire-9
PPI: Patient and public involvement
SAG: Stakeholder Advisory Group
SEND: Special educational needs and disability
WEMWBS: Warwick-Edinburgh Mental Well-being Scale
